# Vascular Smooth Muscle Modulates Endothelial Control of Vasoreactivity via Reactive Oxygen Species Production through Myoendothelial Communications

**DOI:** 10.1371/journal.pone.0006432

**Published:** 2009-07-30

**Authors:** Marie Billaud, Roger Marthan, Jean-Pierre Savineau, Christelle Guibert

**Affiliations:** 1 INSERM, U885, Laboratoire de Physiologie Cellulaire Respiratoire, Bordeaux, France; 2 Université de Bordeaux, Bordeaux, France; Johns Hopkins School of Medicine, United States of America

## Abstract

**Background:**

Endothelial control of vascular smooth muscle plays a major role in the resulting vasoreactivity implicated in physiological or pathological circulatory processes. However, a comprehensive understanding of endothelial (EC)/smooth muscle cells (SMC) crosstalk is far from complete. Here, we have examined the role of gap junctions and reactive oxygen species (ROS) in this crosstalk and we demonstrate an active contribution of SMC to endothelial control of vasomotor tone.

**Methodology/Principal Findings:**

In small intrapulmonary arteries, quantitative RT-PCR, Western Blot analyses and immunofluorescent labeling evidenced connexin (Cx) 37, 40 and 43 in EC and/or SMC. Functional experiments showed that the Cx-mimetic peptide targeted against Cx 37 and Cx 43 (^37,43^Gap27) (1) reduced contractile and calcium responses to serotonin (5-HT) simultaneously recorded in pulmonary arteries and (2) abolished the diffusion in SMC of carboxyfluorescein-AM loaded in EC. Similarly, contractile and calcium responses to 5-HT were decreased by superoxide dismutase and catalase which, catabolise superoxide anion and H_2_O_2_, respectively. Both Cx- and ROS-mediated effects on the responses to 5-HT were reversed by L-NAME, a NO synthase inhibitor or endothelium removal. Electronic paramagnetic resonance directly demonstrated that 5-HT-induced superoxide anion production originated from the SMC. Finally, whereas 5-HT increased NO production, it also decreased cyclic GMP content in isolated intact arteries.

**Conclusions/Significance:**

These data demonstrate that agonist-induced ROS production in SMC targeting EC via myoendothelial gap junctions reduces endothelial NO-dependent control of pulmonary vasoreactivity. Such SMC modulation of endothelial control may represent a signaling pathway controlling vasoreactivity under not only physiological but also pathological conditions that often implicate excessive ROS production.

## Introduction

In vessels, smooth muscle cell (SMC) responses are controlled by endothelial cells (EC) via complex intercellular signaling processes. Regarding vascular tone, the best described interaction is the endothelial-dependent vascular relaxation through the release of nitric oxide (NO) and vasoactive prostanoids [1]. Another pathway is associated with the hyperpolarization of both the EC and SMC and mediated by an endothelium-derived hyperpolarizing factor (EDHF). Whereas EDHF identity is still under debate and may differ among species and vascular segments, there is evidence that EDHF-mediated responses involve epoxyeicosatrienoic acids (EETs) [2], potassium ions and channels [3], reactive oxygen species (ROS) such as hydrogen peroxide (H_2_O_2_) [4] and myoendothelial junctions [5].

In the pulmonary circulation, endothelial control of smooth muscle tone is of critical importance to maintain low pressure and low resistance. In this connection, most of the medical treatments used in pulmonary hypertension (e.g., NO, prostacyclin…) mimic and amplify the physiological control of EC on SMC. For instance, serotonin (5-HT), a potent vasoconstrictor whose concentration is increased in pulmonary arterial hypertension (PAH) [6], [7], acts on both SMC and EC. On one hand, 5-HT can generate NO in pulmonary arterial EC [8]. On the other hand, 5-HT produces ROS in bovine and mice pulmonary arterial SMC and the production of superoxide anion (O_2_^ꜙ^) facilitates 5-HT-induced pulmonary vasoconstriction [9], [10]. O_2_^ꜙ^ can interact with NO to produce peroxynitrite which decreases endothelial NO synthase (eNOS) expression and/or loss of eNOS substrate L-arginine or cofactors tetrahydrobiopterin (BH4) [11]. We thus hypothesized an EC/SMC crosstalk through NO and O_2_^ꜙ^. Gap junctions mediate intercellular communication between EC and SMC (myoendothelial gap junctions) and allow direct exchange of ions and small molecules in various tissues including pulmonary arteries [5], [12]. Whereas gap junctions are expressed in pulmonary artery [13], [14], participate to vascular tone in general [12] and are stimulated by 5-HT in coronary and mesentery SMC [15], no study has been performed on the role of gap junctions in the pulmonary arterial vasoreactivity to 5-HT. Such communication may well participate to the EC/SMC crosstalk. Interestingly, resistance vessels are very important for blood flow regulation and the incidence of myoendothelial gap junctions is higher in resistance than in conduit arteries [16].

In the present study, we have therefore addressed, in small intrapulmonary arteries (IPA), the issue of expression, localization and function of the connexins (Cx) 37, 40 and 43 which are the Cx usually expressed in small vessels. Peptides homologous to the gap 26 and 27 domains of the extracellular loops of Cx 43 (^43^Gap 26) or Cx 37 and 43 (^37-43^Gap 27) respectively, interrupt intercellular communications in a Cx-specific manner and attenuate the calcium and contractile responses to 5-HT. Such effect was reversed by (1) the removal of the endothelium-dependent NO function or (2) the decrease of ROS production. Electronic paramagnetic studies demonstrated that ROS and more specifically O_2_^ꜙ^ was produced by the smooth muscle in response to 5-HT. In the same way, the NO and cyclic GMP content were assessed in response to 5-HT.

Altogether, the findings suggest that 5-HT produces O_2_^ꜙ^ in the smooth muscle and NO in the endothelium. O_2_^ꜙ^ passes through the myoendothelial junctions to decrease endothelial NO production and thus strengthen pulmonary vasoreactivity. This is the first time that a negative control of the endothelial NO function by the smooth muscle is demonstrated. Since ROS production is increased in many cardiovascular diseases, such process could be of great importance under pathological conditions.

## Materials and Methods

### Ethics Statement

All animal work has been conducted according to relevant international and national guidelines in accordance with the recommendations of the Weatherall report, “The use of non-human primates in research”. All the protocols used were approved by our local ethics committee named Comité d'éthique régional d'Aquitaine. The protocol number is AP 2/11/2005.

### Tissue Preparation

*Male Wistar* rats (10–15 weeks old, weighing 300–400 g) were sacrificed using CO_2_ asphyxia according to the animal care and use local committee (Comité d'éthique régional d'Aquitaine – AP 2/11/2005). The left lung was rapidly removed and rinsed in Krebs–HEPES–bicarbonate containing (in mM): 118.4 NaCl, 4.7 KCl, 1.2 MgSO_4,_ 4 NaHCO_3_, 1.2 KH_2_PO_4_, 2 CaCl_2_, 10 N-2-hydroxyethylpiperazine-N'-2-ethanesulfonic acid (HEPES) and 6 D-glucose, pH 7.4 with NaOH. Intrapulmonary arteries with an external diameter of 300–350 µm (IPA) were then dissected free from surrounding connective tissues under binocular control.

### Intracellular Calcium and External Diameter Recording

IPA were cannulated as previously described [17] in an arteriograph (DMT, Denmark). Residual blood was removed by perfusing the vessels with Krebs-HEPES solution, and then vessels were maintained in a no-flow state and held at a constant transmural pressure of 10 mmHg and at a constant temperature of 37°C. Vessels were incubated 1 h in 2 µM Fura-PE3-AM at 37°C and rinsed for 10 min with Krebs-HEPES solution. The arteriograph was then placed on the stage of an inverted epifluorescence microscope (Olympus IX70) equipped with a x10, UPlanApo/0.40 W water-immersion objective (Olympus). The source of excitation light was a xenon arc lamp (175 W), and excitation wavelengths were selected by a wheelfilter (Sutter Instrument Co.) composed of 345 nm and 380 nm filters. Digital images were sampled at 12-bit resolution by a fast-scan, cooled charge-coupled device camera (CoolSNAP fx Monochrome, Photometrics). Preparation was then alternately excited at 345 and 380 nm and emission fluorescence at 510 nm of both excitation wavelengths was imaged every 20 seconds. As previously described, regions of interest were drawn on the vascular wall to determine calcium signal from smooth muscle cells and background was substracted using MetaFluor (Universal Imaging Software) [17]. External diameter of the vessels was measured on these images using MetaMorph (Universal Imaging Software). Contraction was expressed as a percentage related to the percentage of decrease of the initial external diameter.

Either 10 µM or cumulative concentrations of 5-HT (Sigma) were added to the bath, which was bubbled with air. After recording of this first contraction, vessels were washed and pharmacological substances were added both to the bath and to the perfusion solution inside the vessel for one hour. We inhibited gap junction communications through Cx 37 and Cx 43, Cx 40, or Cx 43 with synthetic connexin-mimetic peptides: ^37,43^Gap 27 (SRPTEKTIFII), ^40^Gap 27 (SRPTEKNVFIV) and ^43^Gap 26 (VCYDKSFPISHVR) respectively (Genscript). Specificity of these peptides was checked with an inactive homologous peptide: ^43^Gap 20 (EIKKFKYGC). The role of superoxide anion was investigated with 300 U/ml poly-ethylene glycol-superoxide dismutase (PEG-SOD, Sigma) combined to 600 U/ml poly-ethylene glycol-catalase (PEG-catalase; Sigma). Once substances added, a second contraction with either 10 µM or cumulative concentrations of 5-HT was performed. We performed time-matched controls to check that we can produce two similar cumulative concentration-response curves (CCRC) to 5-HT with one hour of delay between the two CCRC (Figure S1). When indicated, vessels were perfused before contraction protocol with 100 µl of 0.3% 3-((3-cholamidopropyl)diethylammonio)-1-propane sulphonate (CHAPS, Sigma) in order to remove endothelium, as previously described [18].

We checked that such protocol suppressed endothelial function by testing the absence of a relaxation with 10 µM carbamylcholine of 40 mM KCl-induced preconstricted pulmonary arteries (Figure S2). CHAPS on its own had no deleterious effect on the contraction and calcium signal induced by high potassium solution (KCl 40 mM) (Figure S3).

### Isometric Contraction Measurements

Isometric contraction was recorded in intrapulmonary arterial rings with an external diameter of 1.5–2 mm as reported previously [19], [20]. Briefly, mechanical properties were assessed using organ bath and transducer systems, coupled to IOX software (EMKA Technologie). As determined in preliminary experiments, tissues were set at optimal length by equilibration against a passive load of 0.8 g. At the outset of each experiment, K^+^-rich (80 mM) solution, obtained by substituting an equimolar amount of KCl for NaCl from Krebs-HEPES solution, was applied in order to obtain a reference contraction used to normalize subsequent contractile responses. Contractile properties were tested by constructing a CCRC to 5-HT (10 nM to 100 µM) for each ring. When indicated, ^37,43^Gap 27 was incubated during 1 hour before CCRC to 5-HT.

Endothelial function was tested by relaxation with 10 µM carbamylcholine of 0.3 µM phenylephrine-induced preconstricted pulmonary arterial rings. All experiments were performed at 37°C.

### Quantitative RT-PCR

#### RNA Extraction

IPA from one rat was homogenized using 600 µl of Trizol (Invitrogen), then, 120 µl of chloroform (Sigma) was added. The RNA was extracted from the aqueous phase after centrifugation at 15,000 *g* for 15 min. RNA was precipitated in the presence of isopropanol (Sigma) at −20°C overnight. The pure RNA was obtained by centrifugation at 15,000 *g* for 15 min and was washed with 80% ethanol (Sigma). The concentration of RNA was measured spectrophotometrically by GeneQuant RNA/DNA calculator (Amersham Pharmacia). The total RNA (1 µg) was reverse transcribed into cDNA by using AMV reverse transcriptase (Promega), RNase inhibitor, and oligo d(T) as a primer at 42°C for 60 min followed by heating at 94°C for 3 min.

#### Real-time Quantitative Polymerase Chain Reaction (PCR)

Real-time quantitative PCR was performed with a Rotor-Gene 2000 (Corbett Research). Triplicate PCR reactions were assembled in 0.1-ml strip tubes containing cDNA from 10 ng of total RNA, 0.2 µl of 50× Titanium *Taq* DNA Polymerase combined to its buffer (Clontech Laboratories), 1 mM dNTP, each of the appropriate primer (Sigma Genosys; see table 1 for concentrations and sequences), and 0.5× SYBR Green (Molecular Probes).

**Table 1:** Sequences of the primer pairs (S: sense; AS: antisense) for housekeeping genes (GAPDH, HPRT, PLRPO and YWHAZ) and genes of interest (Cx 37, Cx 40 and Cx 43) are shown as well as GenBank accession number, product length, product Tm and concentrations.

Gene	Sequence	GenBank accession number	Product length (pb)	Product Tm (°C)	Concentration (nM)
GAPDH	S: ATTCTACCCACGGCAAGTT AS: CGCCAGTAGACTCCACGACATA	NM_017008	153	89.4	200
HPRT	S: TGTTGGATATGCCCTTGACTA AS: AGATGGCCACAGGACTAGAAC	NM_012583	178	85.6	100
PLRPO	S: AGGTGGGAGCCAGCGAAGC AS: GCAACAGTCGGGTAGCCAATC	NM_022402	208	91.7	100
YWHAZ	S: AGCCGAGCTGTCTAACGAG AS: GCCAAGTAGCGGTAGTAGTCA	NM_013011	291	88.4	100
Cx 37	S: GGTGGCAGAGGACGGTCGTCT AS: CCATGGTCCAGCCGTAGAGA	NM_021654	133	85.3	200
Cx 40	S: GGAAAGAGGTGAACGGGAAG AS: GGGCCTCGAGACATAACAGTT	NM_01280	197	91.3	200
Cx 43	S: TCTGCCTTTCGCTGTAACACT AS: GGGCACAGACACGAATATGAT	NM_012567	117	87.5	200

The PCR was performed under the following conditions: denaturation at 95°C for 15 s, annealing temperature (68–70°C) depending on specific primers for 15 s, and extension at 70°C for 15 s, these steps were repeated during 40 cycles. Data collection was performed after each extension step, at a temperature of at least 3.5°C lower than the melting temperature of the amplicon (generally between 80–85°C) to eliminate non-specific fluorescence signal. PCR negative controls were systematically made by using water instead of cDNA. All specific primers were designed by using the primer analysis software (Oligo 6.6, Molecular Biology Insights). The efficiency of the PCR reactions was always more than 90%. Specificity of the amplified PCR products was checked with melting curve analysis and by electrophoresis analysis on a 2% agarose gel containing SYBR Green.

### Western Blot

IPA from 4 rats were homogenized on ice in lyses buffer containing 1% IGEPAL, 0.5% sodium deoxycholate, 0.1% SDS, 1 mM amino-ethyl-benzenesulfonyl fluoride hydrochloride (AEBSF), 1.5 µM aprotinin and 0.1 mM leupeptin (Sigma-Aldrich). After 10 minutes of centrifugation at 15,000 g, supernatant is reduced in Laemmli buffer and heated at 90°C for 6 min. Protein extract were subjected to electrophoresis on a 10% acrylamid reducing gel, and transferred to polyvinylidene fluoride (PVDF) membranes (Immobilon-P, Millipore). The immunoblots were then incubated using either rabbit anti-Cx 37, rabbit anti-Cx 40 or mouse anti-Cx 43 (Zymed) overnight at 4°C. After incubation with appropriate secondary antibodies coupled to horseradish peroxydase (HRP, Santa Cruz) for 2 h at room temperature, immunoblots were then revealed by enhanced chemiluminescence acquired using Kodak Image Station 4000 MM. Band densities were quantified using GeneTool software (SynGene). Immunoblots were then stripped and revealed with mouse anti-β-actin for 1 h at room temperature.

### Immunofluorescence

IPA were fixed in Formalin (Sigma) for 10 min and then embedded in OCT-Compound, frozen at ­20°C and cut in 10 µm sections with a cryostat. Sections were first incubated in 0.1% Triton X­100 (Sigma) and 1% bovine serum albumin in PBS for 1 h at room temperature, and then in the primary antibodies (same antibodies as the ones used for Western Blot) overnight at 4°C. Sections were then incubated with the appropriate secondary antibodies coupled to Alexa 546 (Molecular Probes) for 2 h at room temperature. Nuclei were labeled with 45 µM Hoechst 33342 (Molecular Probes). Sections were then observed with a laser scanning confocal microscope TE2000 (Nikon) with a x 60, 1.40 NA plan apochromat oil-immersion objective. Excitation was obtained with a diode laser at 408 nm to observe nuclei, an argon laser at 488 nm to observe autofluorescence of the internal elastic lamina and a helium-neon laser at 543 nm to observe Cx labeling. The emitted light was filtered as appropriate: 450±35 nm for nuclei (blue), 515±30 nm for internal elastic lamina (green), and 605±75 nm for Cx labeling (red).

### Dye Transfer

IPA were mounted in an arteriograph as previously described (see Intracellular calcium and external diameter recording). The vessels were allowed to equilibrate and pressurized at 10 mmHg for 30 min, and then lumen was loaded with 10 µM carboxyfluorescein-AM (Molecular Probes) for 30 min before washout with Krebs-HEPES for 30 min. Where indicated, vessels were perfused with 300 µM ^37,43^Gap 27 for 1 h before carboxyfluorescein loading. Vessels were subsequently removed from the arteriograph and fixed in Formalin for 20 min at room temperature before cryopreservation in OCT compound at −20°C. Cryosections (10 µm thick) were prepared and mounted in Fluorescent mounting medium (Dako) and imaged with laser scanning confocal microscope TE2000 (Nikon) with a x 60, 1.40 NA plan apochromat oil-immersion objective. Preparation was excited with an argon laser at 488 nm, and fluorescence emission was filtered at 515 nm±30.

### Electron Paramagnetic Resonance (EPR) Recordings

Intrapulmonary arteries with an external diameter of 0.3–2 mm were used for EPR studies and cGMP measurements.

#### Superoxide Anion Spin Trapping

Pulmonary arteries were incubated in the spin trap solution containing 500 µM 1­hydroxy-3-methoxycarbonyl-2,2,5,5-tetramethylpyrrolidin (CMH, Noxygen), 25 µM deferoxamine (Sigma) and 5 µM *N,N*-diethyldithiocarbamate (DETC, Sigma) in Krebs-HEPES at 37°C for 45 min. 5-HT 0.1 mM was added during the spin trap incubation; PEG-SOD 300 U/mL was incubated during 45 min before spin trap incubation. Reaction was stopped by freezing the sample in liquid nitrogen. Samples were then analyzed by EPR spectrometry on a tabletop x-band spectrometer miniscope (MS200, Magnettech). Spectra of the oxidized product of CMH (CM•) were recorded at 77°K using a flask Dewar. Acquisition parameters were as followed: Bo Field: 3341±150 G, microwave power: 10 dB, amplitude modulation: 5 G, sweep time: 60 sec, gain: 300 and 3 scans [21]. Signals were quantified by measuring the total amplitude, after correction of baseline and normalized to the protein quantity of the sample in mg/ml.

#### NO Spin Trapping

Detection of NO production was performed using the technique with Fe^2+^-DETC as spin trap. IPA were placed in 0.5 ml of Krebs-HEPES and then treated with 0.5 ml of colloid Fe(DETC)_2_ as previously described and incubated for 45 min at 37°C [22]. When indicated, 0.1 mM 5-HT was added during Fe(DETC)_2_ incubation. NO measurements were performed at 77°K using EPR spectrometry. Instrument settings were: Bo Field: 3285±80 G, microwave power: 10 dB, amplitude modulation: 7 G, sweep time: 150 sec, gain: 900 and 3 numbers of scan. Signals were quantified by measuring the total amplitude, after correction of baseline and normalized to the protein quantity of the sample in mg/ml.

### cGMP Measurements

Intrapulmonary arteries were incubated in Krebs-HEPES with or without 5-HT 0.1 mM and with or without PEG-SOD + PEG-catalase (300 U/ml and 600 U/ml respectively) at 37°C for 15 min, cooled in liquid nitrogen and then stored at −80°C. Arteries were homogenized in ice-cold trichloroacetic acid (5%, Sigma) to extract cGMP. cGMP content was assayed as described in the procedure of an ELISA kit obtained from Cayman Chemical & Co. cGMP level was normalized to tissue protein content in mg/ml.

### Statistical Analysis

All results are expressed as means±SEM, n indicates the number of IPA for the calcium and contractile studies, dye transfer and immunofluorescence, the number of rats for quantitative RT-PCR, EPR and cGMP measurements, and the number of experiments from 3 pools of rats (4 rats per pool) for Western Blot. Cumulative concentration response curves to 5-HT were fitted to the logistic equation with Origin 6.0 software to determine the maximum effect of 5-HT for each experiment, and a non parametric test for paired samples (Wilcoxon test) was performed on the maximum effect of 5-HT with or without drug. Quantitative RT-PCR results were analyzed with the GeNorm method [23]. Statistical analyses were performed on all other data using a non parametric test for unpaired samples (Mann-Witney test). Values of *P*<0.05 were considered significant.

## Results

### Connexins 37, 40 and 43 are expressed and functional in the pulmonary arterial wall

**Figure 1:**
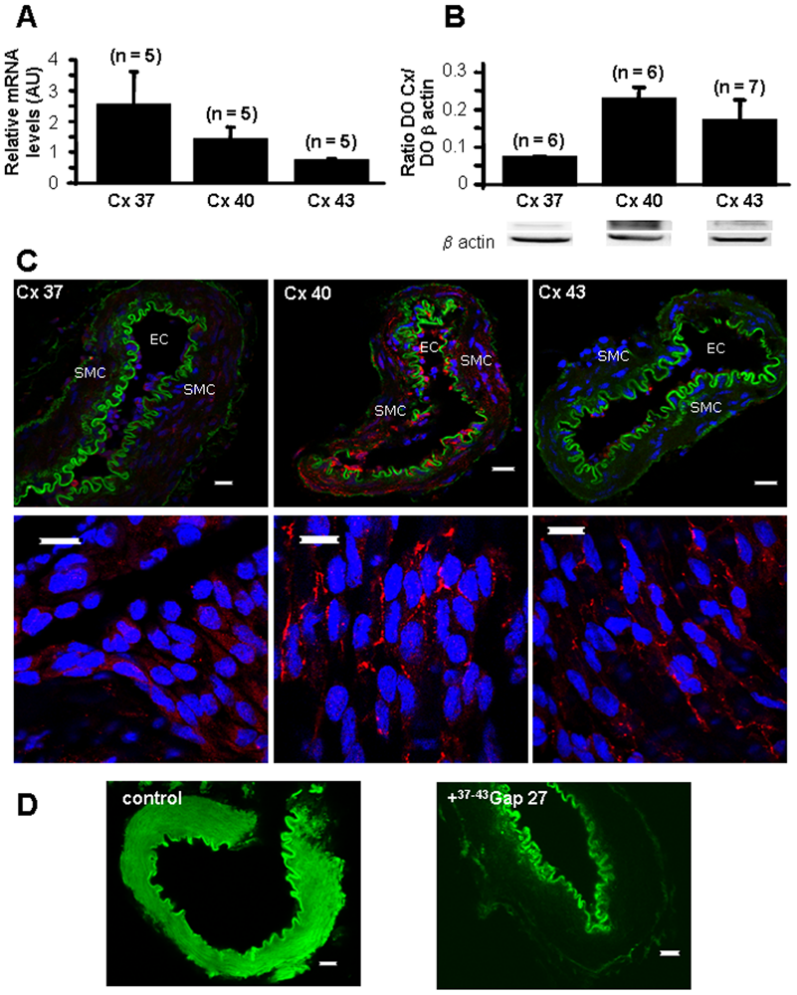
Expression and function of connexins 37, 40 and 43. A shows mean values±S.E.M. of relative mRNA expression levels measured by quantitative RT-PCR. B shows protein expression measured by Western Blot and normalized to β actin expression level. C shows immunofluorescent labeling demonstrating the presence of Cx (red) 37 (left), 40 (middle) and 43 (right). Green shows the autofluorescent signal of the vessels and blue the nuclei (C). C bottom are pictures of the endothelial side of opened vessels. SMC means smooth muscle cells and EC means endothelial cells. D shows diffusion of a green fluorescent dye from the endothelium to the smooth muscle via cell-to-cell communications in basal conditions (left) and such diffusion was blocked following one hour incubation with 300 µM ^37-43^Gap 27 (right). Scale bars are 15 µm. n indicates the number of rats for quantitative RT-PCR and the number of experiments performed for Western Blot.

Although very few studies have been performed with controversial results in pulmonary arteries, the three Cx 37, 40 and 43 have been observed in pulmonary arteries [13], [14]. Quantitative RT-PCR and Western Blot experiments evidenced the presence of the mRNA and proteins for the three Cx 37, 40 and 43 in small intrapulmonary arteries (IPA) (Figure 1A and B). Immunofluorescent labeling studies confirmed the presence of the Cx 37, 40 and 43 proteins (Figure 1C). The autofluorescence of the external and internal elastic lamina in green has been previously observed in various vessels [24], [25] and is useful to delimit the smooth muscle layers from the endothelial cells and the adventitia. Some punctate labeling was observed for the Cx 37, 40 and 43 in the endothelium and similar labeling was observed in the smooth muscle for the Cx 37 and 40 (Figure 1C). Punctate staining is characteristic of connexins labeling and has been previously shown in various tissues including vessels [4], [24], [25]. Since (1) Cx 37 and 43 are expressed in IPA, (2) they have a functional role in both the calcium and contractile signal and (3) they are localized in the endothelium, we then addressed the functional role of the Cx 37 and 43 in the myoendothelial communications and/or the communications between endothelial cells. After loading endothelial cells with a fluorescent dye (carboxyfluorescein), the dye diffused to the smooth muscle cells in control conditions (Figure 1D left) whereas it stayed in the endothelium when the vessels were pretreated with ^37,43^Gap 27 300 µM (Figure 1D right).

Altogether, it can be concluded that the myoendothelial communications are functional and involve the Cx 43 and/or Cx 37.

### Vasoreactivity to 5-HT is controlled by gap junctions and endothelium

**Figure 2:**
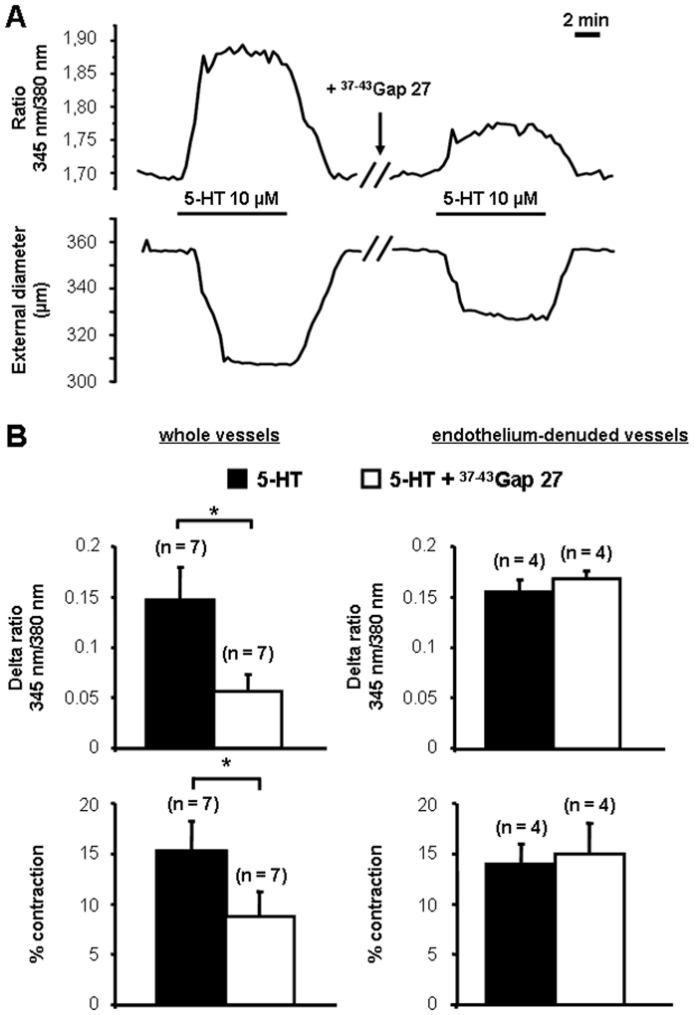
Myoendothelial and/or endothelial gap junctions participate to the calcium and contractile responses to 5-HT. Typical traces of simultaneous recordings of intracellular calcium (top panel) and contraction (bottom panel) induced by 5­HT 10 µM are shown in A. 300 µM ^37-43^Gap 27 was introduced into the bath where indicated. B shows the mean values±S.E.M. for the effect of ^37-43^Gap 27 on both the amplitude of the calcium response to 5-HT 10 µM (top) and the percentage of contraction (bottom) in whole vessels (left) and in endothelium-denuded vessels (right). Black columns show the responses to 5-HT in the absence of ^37-43^Gap 27 and white columns are the same responses in the presence of ^37-43^Gap 27. Data are expressed as a delta ratio (345 nm/380 nm) for calcium signal and the percentage of contraction is related to the percentage of decrease of the initial external diameter. n indicates the number of vessels studied. * indicates a significant difference when *P*<0.05.

We then examined if gap junctions had any influence on (1) the intracellular calcium concentration in smooth muscle and (2) the contraction in IPA. Consequently, we checked if any of these connexins had any role in the simultaneously recorded calcium and contractile signals in response to the agonist 5-HT in IPA. Incubation of the vessels with ^40^Gap 27 300 µM, the Cx-mimetic peptide used as a specific blocker of the Cx 40, had no effect on both calcium and contractile signals (Figure S4). In contrast, incubation with ^37,43^Gap 27 300 µM, the Cx-mimetic peptide used as a specific blocker of the both Cx 37 and 43, strongly decreased both calcium and contractile signals to 5-HT (Figure 2A and B left). Incubation with ^43^Gap26 300 µM, the specific blocker of the Cx 43 alone, had similar effects on calcium and contractile signals to those of the ^37,43^Gap 27 (Figure S5) suggesting that the Cx 43 is involved in the response to 5-HT. Since ^43^Gap 20 is a peptide homologous to the intracellular loop of the Cx 43, it can be used as an inactive analog of the Gap 27 specific of the Cx 43 [26]. We observed that ^43^Gap 20 300 µM had no effect on the calcium and contractile signals to 5-HT confirming the specificity of these Cx-mimetic peptides (Figure S6).

Since gap junctions are localized in between smooth muscle cells and/or in between endothelial cells and/or in between smooth muscle and endothelial cells (myoendothelial junctions), we removed the endothelium of the vessels to discriminate the role of these different types of communications. The blocking effect of the ^37,43^Gap 27 300 µM was absent in endothelium-denuded vessels (Figure 2B right) suggesting that the myoendothelial and/or the endothelial junctions are important for the calcium and contractile signals to 5-HT.

Isometric tension recordings showed that whereas ^37,43^Gap 27 300 µM blocked the CCRC to 5­HT (0.01–100 µM), it had no effect on the CCRC to phenylephrine (0.1 nM–3 µM), to endothelin-1 (0.1–5000 nM) and to high potassium solutions (4.7–100 mM, Figure S7). Moreover, in systemic vessels such as renal arteries, the CCRC to 5-HT were identical in the presence or in the absence of ^43^Gap 26 300 µM (Figure S8A) whereas in aorta, ^43^Gap 26 300 µM partially inhibited the CCRC to 5-HT (Figure S8B).

### NO and O_2_^ꜙ^ are involved and interconnected in the calcium and contractile responses to 5­HT

**Figure 3:**
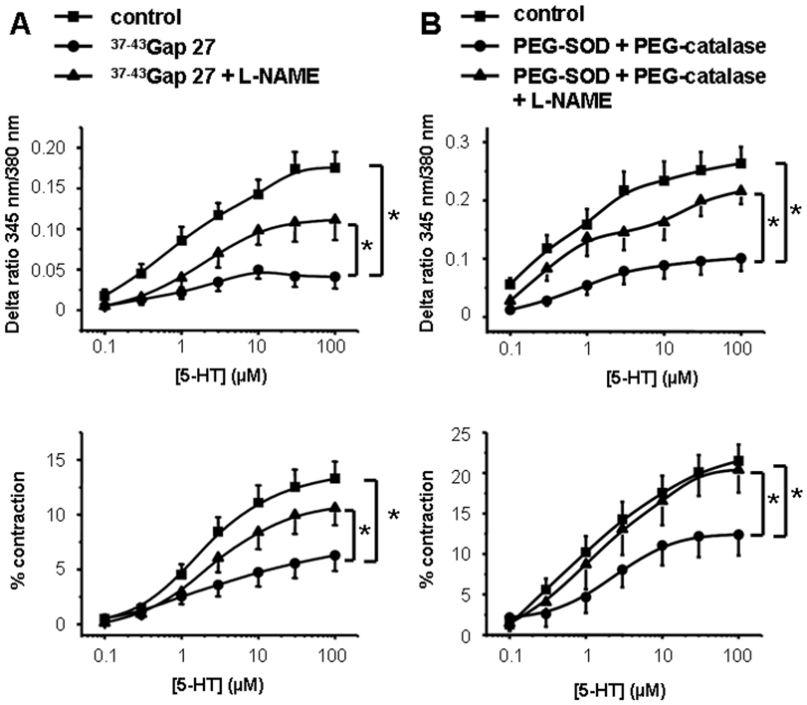
Contribution of gap junctions, O_2_^ꜙ^ and NO in the calcium and contractile responses to 5-HT. Concentration response curves to 5-HT (0.1–100 µM) were performed simultaneously on the calcium signal (top panels) and the contraction (bottom panels). Data points are means±S.E.M. A, 300 µM ^37-43^Gap 27 strongly blocked the calcium and contractile signals to 5-HT (black circles, n = 7 vessels). B, PEG-SOD (300 U/ml) and PEG-catalase (600 U/ml) also inhibited the calcium and contractile responses to 5-HT (black circles, n = 7 vessels). Both ^37-43^Gap 27 and PEG-SOD plus PEG-catalase effects were reversed by L-NAME 100 µM (A and B respectively, black triangles, n = 6 and 9 vessels respectively). Black squares are control dose-response curves to 5-HT (n = 13–16 vessels). * indicates a significant difference when *P*<0.05.

**Figure 4:**
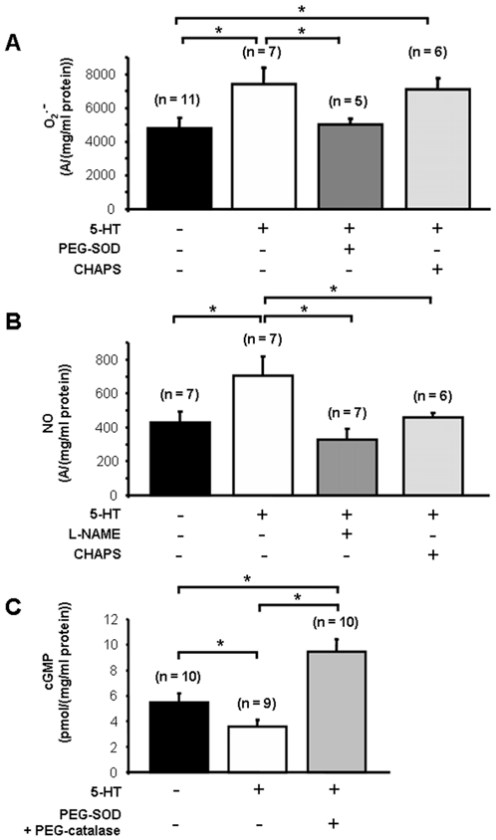
Production of O_2_^ꜙ^, NO and cyclic GMP by 5-HT. A, O_2_^░^ production was measured by EPR spectrometry in the absence or in the presence of 5-HT 100 µM (black column and white column respectively). The production of O_2_^░^ by 5-HT was reduced by PEG-SOD (A, grey column) whereas it was unchanged when vessels were endothelium-denuded by CHAPS (A, light grey column). B, EPR spectrometry also showed an increase in NO production by 5-HT 100 µM (white column) and an absence of NO production by 5-HT 100 µM when vessels were endothelium-denuded by CHAPS (light grey column) or treated with L-NAME 100 µM (grey column). C, as measured by Elisa kit, basal cyclic GMP content (black column) was decreased by 5-HT (white column) whereas it was increased when PEG-SOD and PEG-catalase were added to 5-HT (light grey column). Data from EPR spectrometry are expressed as a ratio of the amplitude of the pic (A) out of the protein concentration of each pool of vessels in mg/ml. cGMP contents are expressed as a ratio of the quantity of cGMP in picomoles out of the protein concentration of each pool of vessels in mg/ml. Values are means±S.E.M. and n indicates the number of rats tested. * indicates a significant difference when *P*<0.05.

Since NO is a major element contributing to the endothelial control of vasoreactivity, we checked the effect of L-NAME, a NO synthase inhibitor, on the blocking effect of ^37,43^Gap 27 previously observed on the simultaneously recorded calcium and contractile signals to 5-HT. L-NAME 100 µM reversed the blocking effect of ^37,43^Gap 27, 300 µM, on both the intracellular calcium concentration and contraction to 5-HT (Figure 3A) whereas L-NAME 100 µM alone had no effect (Figure S9) confirming the importance of NO in the endothelial control of the vasoreactivity through gap junctions. We hypothesized that O_2_^ꜙ^ production by 5-HT could account for a reduction of the endothelial NO production leading to an increased contraction since O_2_^ꜙ^ is a small element which likely passes the membranes through gap junctions and is able to scavenge NO and/or reduce its production thus decreasing its relaxant effect [11], [27]. Indeed, poly-ethylene glycol-superoxide dismutase (PEG-SOD) 300 U/ml and poly-ethylene glycol-catalase (PEG-catalase) 600 U/ml, two substances which catabolise O_2_^ꜙ^, reduced calcium and contractile responses to 5-HT and such effect was reversed by L-NAME 100 µM (Figure 3B). Electron paramagnetic resonance (EPR) recordings demonstrated that 5-HT produced O_2_^ꜙ^ (Figure 4A). This O_2_^ꜙ^ production was prevented by PEG-SOD 300 U/ml and unchanged when the endothelium was removed by CHAPS 0.3% (Figure 4A). We checked that CHAPS 0.3% had no effect on its own on basal O_2_^ꜙ^ levels (Figure S10). Consequently, 5-HT produced O_2_^ꜙ^ originating from smooth muscle. Moreover, we also directly detected the production of NO by 5-HT with EPR experiments (Figure 4B). When the vessels were endothelium-denuded by CHAPS or treated with L-NAME 100 µM, the production of NO by 5-HT was abolished (Figure 4B) showing respectively that 1) NO produced by 5-HT originates from the endothelium and 2) the product detected by EPR is linked to NO synthases.

**Figure 5:**
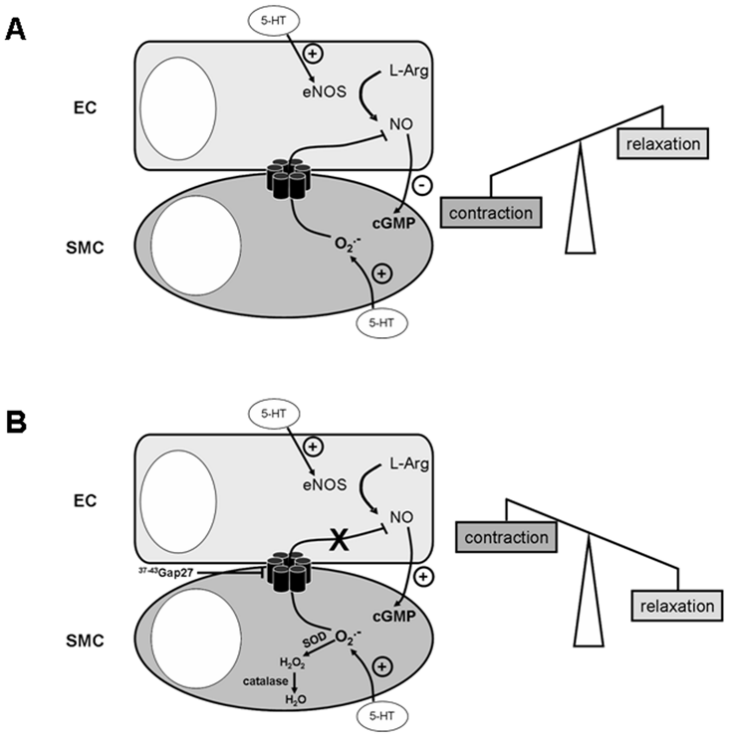
Proposed mechanism for the negative feedback of smooth muscle on endothelial relaxant control of vasoreactivity. 5-HT can act on both SMC and EC. Calcium increase in SMC in response to 5-HT leads to contraction whereas in EC it stimulates NO synthase and leads to NO production. NO will stimulate guanylate cyclase which will increase cGMP production in SMC and then induce relaxation. In normal conditions, 5-HT produces O_2_^░^ in SMC. O_2_^░^ passes through myoendothelial gap junctions and scavenges NO in EC thus decreasing cGMP content in SMC. Consequently, 5-HT effect on SMC prevails over 5-HT effect on EC and will induce a contraction (A). If (1) O_2_^░^ is degraded by SOD and catalase, and/or (2) gap junctions are blocked, NO scavenging by O_2_^░^ in EC will be prevented. Consequently, NO and cGMP levels will be increased thus inducing a relaxant effect leading to a lower contraction to 5-HT in the whole vessels (B).

Finally, since, on one hand, 5-HT produced NO that induced relaxation via cGMP production in SMC and, on the other hand, it also produced O_2_^ꜙ^ that antagonized NO, we measured the cGMP content in order to determine if the overall result was in favor of a relaxant or a contractile effect. Whereas 5-HT significantly reduced the basal cGMP content, in the additional presence of PEG-SOD 300 U/ml and PEG-catalase 600 U/ml, it did increase the cGMP content (Figure 4C). This result indicates that O_2_^ꜙ^ produced by 5-HT in SMC decreases the NO produced by 5-HT in EC, thus decreasing cGMP level leading to an increased vascular contraction (Figure 5A). To the best of our knowledge, this is the first description of a negative feedback of smooth muscle on endothelial relaxant control of vasoreactivity.

## Discussion

Our findings provide the first direct evidence of a smooth muscle negative feedback on NO-dependent endothelial vasodilatation thus maintaining some contraction in vessels under physiological pressures. In particular, we showed that the Cx 43 is expressed and functional in IPA and is necessary to develop a sustained agonist-mediated contraction in an integrated vascular model. Our data also support that such process involves agonist-induced production of O_2_^ꜙ^ in the smooth muscle and NO in the endothelium. O_2_^ꜙ^, via myoendothelial junctions, reduces NO-induced cGMP production in smooth muscle and the resulting vessel relaxation (Figure 5).

In small vessels, gap junctions are usually mainly involved in spontaneous oscillations of membrane potential, intracellular calcium concentration and contraction [24], [28], [29]. Gap junctions have also been shown to be largely implicated in the mediators- and endothelium-related relaxation [5], [30]. Few studies have addressed the role of gap junctions in agonist-induced contraction and the results vary according to studies. Indeed, gap junction blockers either increase or decrease the contraction and intracellular calcium to phenylephrine in rat mesenteric arteries [24], [29]. Interestingly, arteries that express Cx 43 to a high degree, such as the rat aorta, are more sensitive to adrenergic agonists than those expressing Cx 43 at a low level such as the caudal artery [30], [31]. Altogether, these results are consistent with our study namely stimulation of gap junctions, and especially Cx 43, is important to strengthen the agonist-related vascular contraction. However, the mechanisms involved in this regulation of the vascular tone by gap junctions remain elusive.

Gap 26 and Gap 27 connexin-mimetic peptides have been largely used as specific blockers of the gap junctions [24], [32]. In our hands, ^37-43^Gap 27 and ^43^Gap 26, firstly, inhibited the calcium and contractile signals to 5-HT whereas the inactive analog ^43^Gap 20 had no effect and, secondly, ^37-43^Gap 27 prevented the diffusion of a fluorescent dye from the endothelial cells to the smooth muscle cells demonstrating the specificity of these blockers on the connexins.

In vessels, Ca^2+^ and inositol 1, 4, 5 trisphosphate (IP_3_) are intercellular messengers that pass through the intercellular communications and especially the myoendothelial communications [28], [33]. In a model of cocultured endothelial and smooth muscle cells, both IP_3_ and calcium originating in the vascular SMC appear to cross myoendothelial communications to induce a secondary calcium increase in endothelial cells whereas only calcium, and not IP_3_, originating from EC crosses myoendothelial junctions to generate a calcium dependent response in the vascular SMC [33]. In an integrated model of pressurized mesenteric arteries, such regulation by surrounding SMC via myoendothelial gap junctions, even under basal conditions, has also been described [28]. However, under those conditions, IP_3_ from SMC increases calcium in EC and, consequently, enhances endothelium-related relaxation rather than contraction. In our study, the negative control of this secondary endothelial calcium signal linked to relaxation allows the smooth muscle to produce a sustained rather than a transient contraction.

There are some lines of evidence from the literature that O_2_^ꜙ^ (1) is produced by 5-HT in mice and bovine pulmonary arterial SMC and (2) participates to the contraction or proliferation of SMC [10], [34]. Although, Liu and Folz [10] used an integrated model of mouse intrapulmonary arteries, they did not address the role of gap junctions in the contraction to 5-HT. In pulmonary artery, 5-HT is well known to induce contraction via stimulation of 5-HT receptors and SMC proliferation via stimulation of 5-HT transporter and/or 5-HT receptors [6]. O_2_^ꜙ^ production in mice intrapulmonary arteries is insensitive to GR 127935, a selective inhibitor of 5-HT1B/D receptor and it is sensitive to 5-HT transporter in bovine pulmonary arterial SMC [10], [35]. We did not address the role of 5-HT transporter and/or receptor in our model as this issue deserves future study. In the same way, Liu and Folz suggested that O_2_^ꜙ^ was produced via a NADPH oxidase pathway [10] and this hypothesis could apply to our study.

O_2_^ꜙ^ is increased in several models of hypertension including pulmonary arterial hypertension [36], [37]. Moreover, it has been observed that increased contraction to 5-HT in aortic strips of hypertensive rats is inhibited by the blockade of the gap junctions [38]. Finally, pulmonary arterial hypertension is linked to an endothelial dysfunction [39]. These data suggest that the process we propose, namely the negative regulation of the endothelial relaxation by the smooth muscle, could also be of great interest in vascular pathophysiological conditions.

In conclusion, we evidenced vascular smooth muscle can negatively control the endothelial relaxation in order to produce a sustained contraction in small vessels at physiological pressures. This control involves O_2_^ꜙ^, myoendothelial junctions and regulation of endothelial NO function. In that respect, modifications of this process could potentially be involved in vascular pathologies and need to be further investigated.

## Supporting Information

Figure S1 Reproducibility of two cumulative concentration-response curves to 5-HT on the same vessel. Two CCRC to 5-HT (0.1–100 µM) recorded on the same vessel with a delay of one hour in between the two curves were similar for both calcium signal (top) and contraction (bottom). Black squares indicate the first CCRC and the black circles indicate the second CCRC. Data are means±S.E.M. for 8 vessels and are expressed as a delta ratio (345 nm/380 nm) for calcium signal and a percentage of contraction (top and bottom respectively). The percentage of contraction is related to the percentage of the initial external diameter.
(0.30 MB TIF)

Figure S2 Effect of endothelium removal on the relaxant effect of carbamylcholine. Intrapulmonary arteries were preconstricted with high potassium solution (KCl 40 mM) and then stimulated with 10 µM carbamylcholine. Calcium and contractile signals were simultaneously recorded (top and bottom respectively). The experiments were performed in control vessels (black column) and in vessels whose endothelium has been denuded with CHAPS (white column). Data are means±S.E.M. and are expressed as a percentage of the delta ratio (345 nm/380 nm) or a percentage of the contraction in response to carbamylcholine 10 µM from preconstricted vessels with KC1 40 mM (top and bottom respectively). n indicates the number of vessels tested. * indicates a significant difference when P<0.05.
(0.21 MB TIF)

Figure S3 Effect of endothelium removal on the calcium and contractile responses to high potassium solution. The calcium and contractile signals simultaneously recorded in response to high potassium solution (KCl 40 mM) were similar in control vessels (black column) and in vessels whose endothelium has been denuded with CHAPS (white column). Data are means±S.E.M. and are expressed as a delta ratio (345 nm/380 nm) for calcium signal and a percentage of contraction (top and bottom respectively). n indicates the number of vessels tested.
(0.19 MB TIF)

Figure S4 Effect of 40Gap 27 on the calcium and contractile signals in response to 5-HT. The calcium and contractile signals were simultaneously recorded in response to 5-HT 10 µM in the absence (black column) or in the presence of 300 µM 40Gap 27, the Cx-mimetic peptide targeted against Cx 40 (white column). Data are means±S.E.M. and are expressed as a delta ratio (345 nm/380 nm) for calcium signal and a percentage of contraction (top and bottom respectively). n indicates the number of vessels tested.
(0.18 MB TIF)

Figure S5 Effect of 43Gap 26 on the calcium and contractile signals in response to 5-HT. Cumulative concentration-response curves to 5-HT (0.1–100 µM) were performed in the absence or in the presence of 300 µM 43Gap 26, the Cx-mimetic peptide targeted against Cx 43 (black squares and circles respectively). Data are means±S.E.M. for 7 vessels and are expressed as a delta ratio (345 nm/380 nm) for calcium signal and a percentage of contraction (top and bottom respectively). * indicates a significant difference when P<0.05.
(0.30 MB TIF)

Figure S6 Effect of 43Gap 20 on the calcium and contractile signals in response to 5-HT. CCRC to 5-HT (0.1–100 µM) were performed in the absence or in the presence of 300 µM 43Gap 20, an inactive analog of the Cx-mimetic peptide targeted against Cx 43 (black squares and circles respectively). Data are means±S.E.M. for 7 vessels and are expressed as a delta ratio (345 nm/380 nm) for calcium signal and a percentage of contraction (top and bottom respectively).
(0.30 MB TIF)

Figure S7 Effect of 37-43Gap 27 on the contractile responses to 5-HT, phenylephrine, endothelin-1 and high potassium solutions. Isometric tension measurements were recorded on intrapulmonary arterial rings in response to cumulative concentrations of 5-HT (A) or phenylephrine (B) or endothelin-1 (C) or in response to increasing concentrations of potassium (KCl 4.7 - 100 mM) (D). Contractions were recorded in the absence (black squares) or in the presence (black circles) of 300 µM 37-43Gap 27. Data are means±S.E.M. for 7 - 21 vessel rings and are expressed as a percentage of the contraction to high potassium solution (KCl 80 mM). * indicates a significant difference when P<0.05.
(0.41 MB TIF)

Figure S8 Effect of 43Gap 26 on the contractile responses to 5-HT in renal arteries and aorta. Isometric tension measurements were recorded on arterial rings in response to cumulative concentrations of 5-HT in renal arteries (A) or aorta (B). Contractions were recorded in the absence (black squares) or in the presence (black circles) of 300 µM 43Gap 26. Data are means±S.E.M. for 8 - 16 vessel rings and are expressed as a percentage of the contraction to high potassium solution (KCl 80 mM). * indicates a significant difference when P<0.05.
(0.29 MB TIF)

Figure S9 Effect of L-NAME on the simultaneously recorded calcium and contractile signals in response to 5-HT. Cumulative concentration-response curves to 5-HT (0.1–100 µM) were performed in the absence or in the presence of L-NAME 100 µM, a NO synthase inhibitor (black squares and circles respectively). Data are means±S.E.M. for 6 vessels and are expressed as a delta ratio (345 nm/380 nm) for calcium signal and a percentage of contraction (top and bottom respectively).
(0.29 MB TIF)

Figure S10 Effect of endothelium removal and xanthine plus xanthine oxidase treatment on superoxide anion production. O2• was measured by using CMH spin trapping method and EPR spectrometry on control vessels (black column), vessels whose endothelium was denuded with CHAPS 0.3% (white column, top panel) and vessels treated with xanthine 50 µM plus xanthine oxidase 0.02 U/ml (white column, bottom panel). Data are means±S.E.M. and are expressed as a ratio of the amplitude of the pic (A) out of the protein concentration of each pool of vessels in mg/ml. n indicates the number of vessels tested.
(0.24 MB TIF)
