# Supplementary figures and images for: Vascular Smooth Muscle Modulates Endothelial Control of Vasoreactivity via Reactive Oxygen Species Production through Myoendothelial Communications
Source: PLoS One. 2009 Jul 30;4(7):e6432. doi: 10.1371/journal.pone.0006432 (PMC2713830; doi:10.1371/journal.pone.0006432)

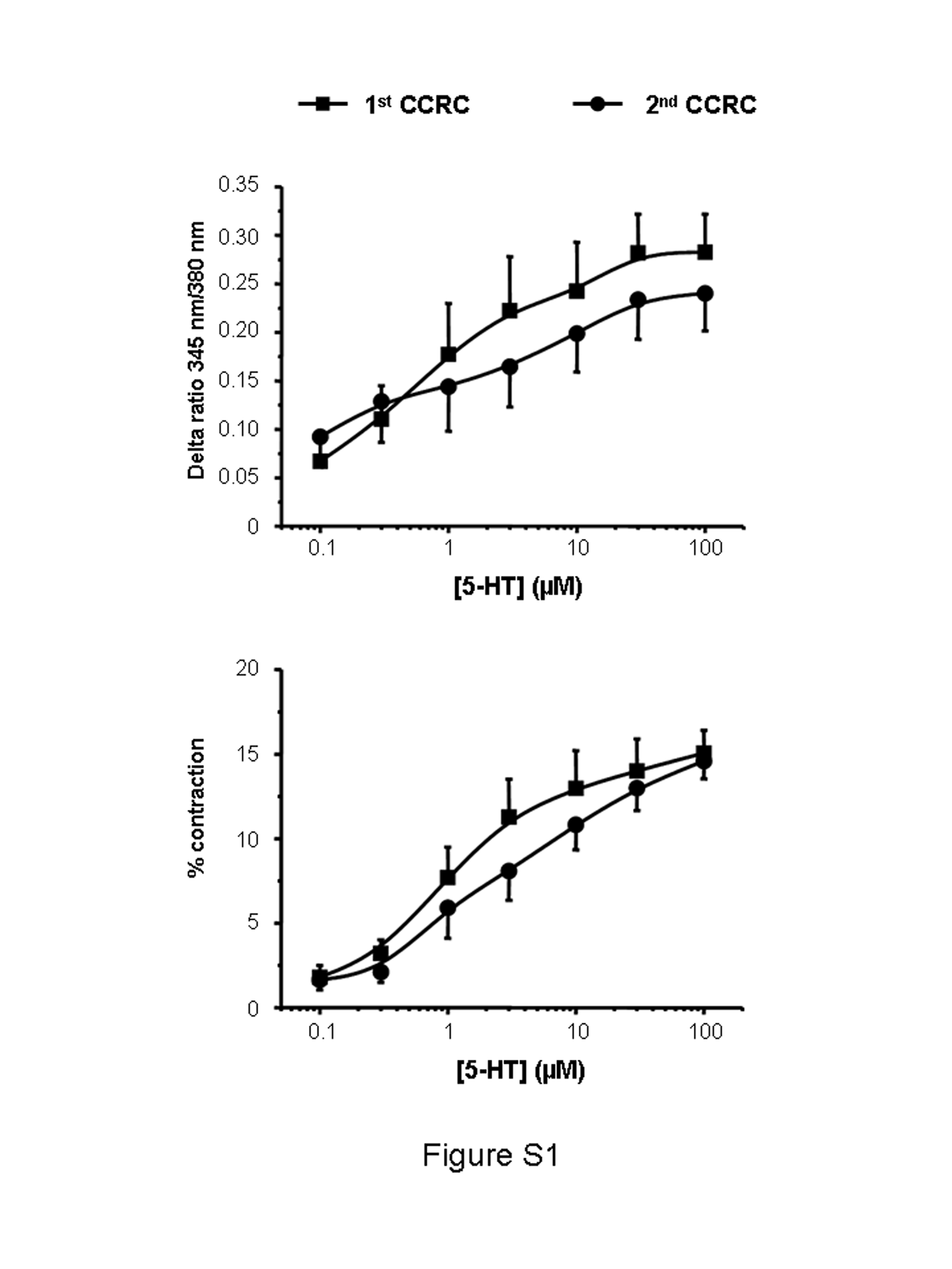

Supplement: Figure S1 — Reproducibility of two cumulative concentration-response curves to 5-HT on the same vessel. Two CCRC to 5-HT (0.1–100 µM) recorded on the same vessel with a delay of one hour in between the two curves were similar for both calcium signal (top) and contraction (bottom). Black squares indicate the first CCRC and the black circles indicate the second CCRC. Data are means±S.E.M. for 8 vessels and are expressed as a delta ratio (345 nm/380 nm) for calcium signal and a percentage of contraction (top and bottom respectively). The percentage of contraction is related to the percentage of the initial external diameter. (0.30 MB TIF) [file pone.0006432.s001.tif]

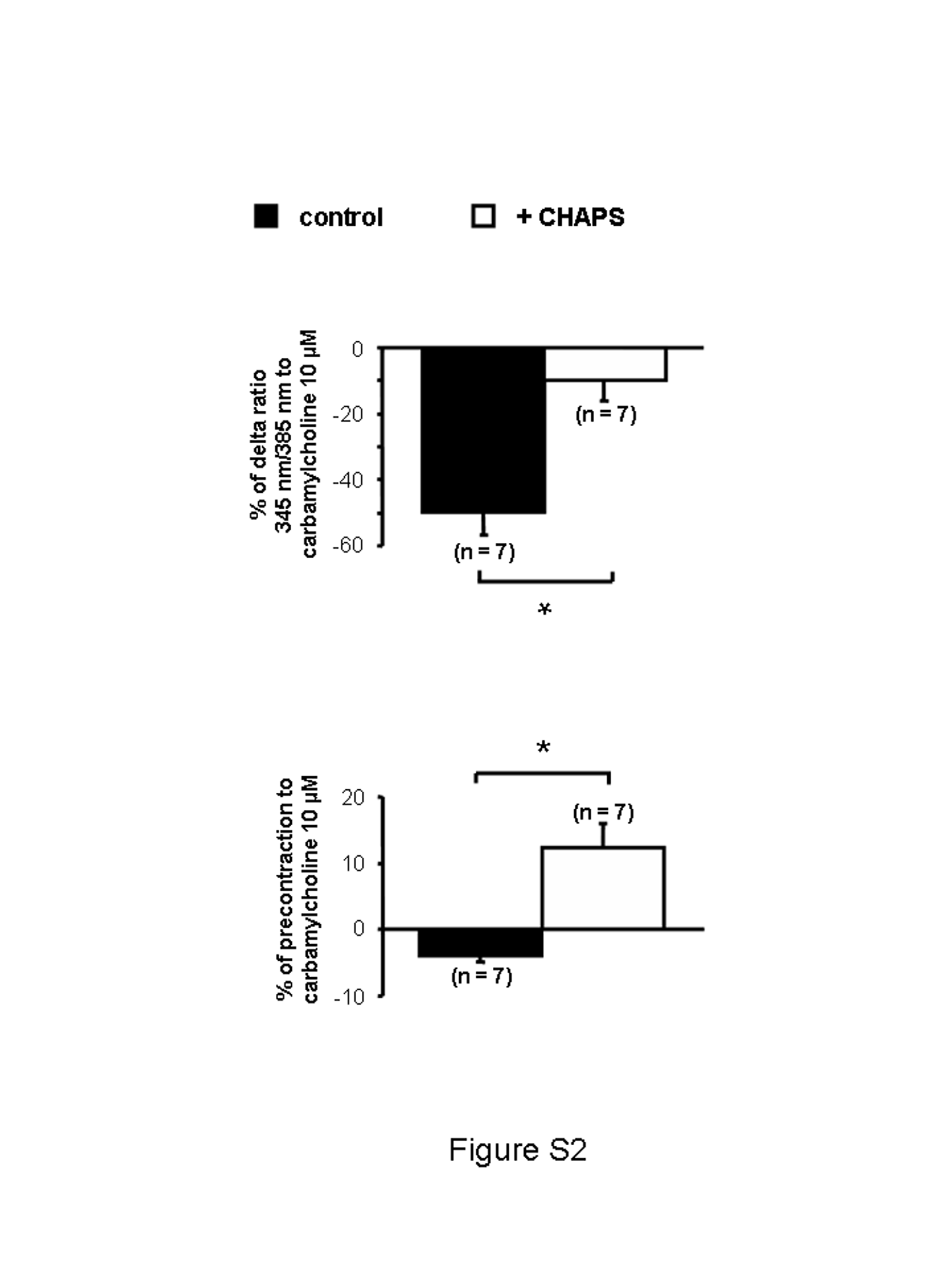

Supplement: Figure S2 — Effect of endothelium removal on the relaxant effect of carbamylcholine. Intrapulmonary arteries were preconstricted with high potassium solution (KCl 40 mM) and then stimulated with 10 µM carbamylcholine. Calcium and contractile signals were simultaneously recorded (top and bottom respectively). The experiments were performed in control vessels (black column) and in vessels whose endothelium has been denuded with CHAPS (white column). Data are means±S.E.M. and are expressed as a percentage of the delta ratio (345 nm/380 nm) or a percentage of the contraction in response to carbamylcholine 10 µM from preconstricted vessels with KC1 40 mM (top and bottom respectively). n indicates the number of vessels tested. * indicates a significant difference when P<0.05. (0.21 MB TIF) [file pone.0006432.s002.tif]

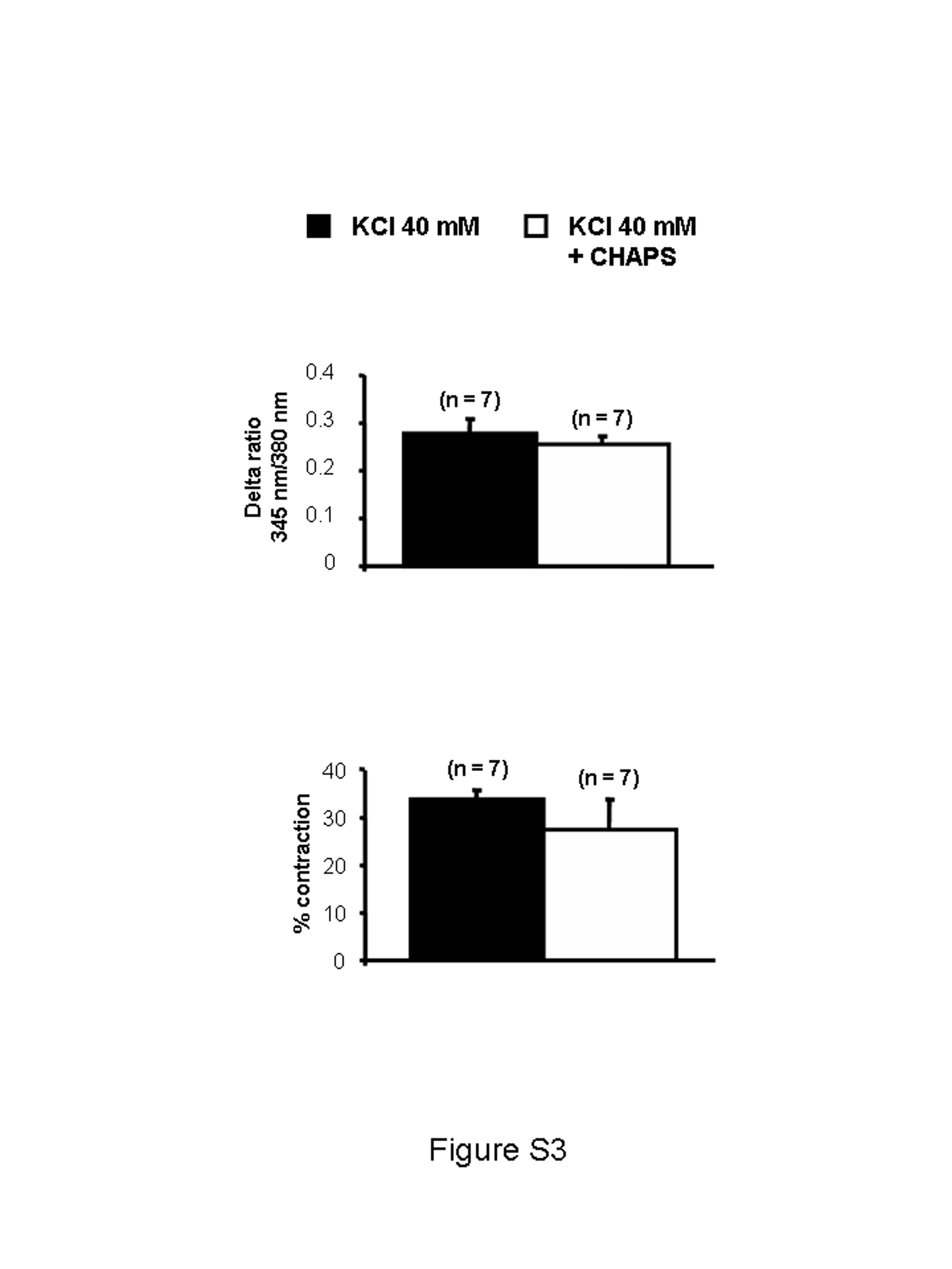

Supplement: Figure S3 — Effect of endothelium removal on the calcium and contractile responses to high potassium solution. The calcium and contractile signals simultaneously recorded in response to high potassium solution (KCl 40 mM) were similar in control vessels (black column) and in vessels whose endothelium has been denuded with CHAPS (white column). Data are means±S.E.M. and are expressed as a delta ratio (345 nm/380 nm) for calcium signal and a percentage of contraction (top and bottom respectively). n indicates the number of vessels tested. (0.19 MB TIF) [file pone.0006432.s003.tif]

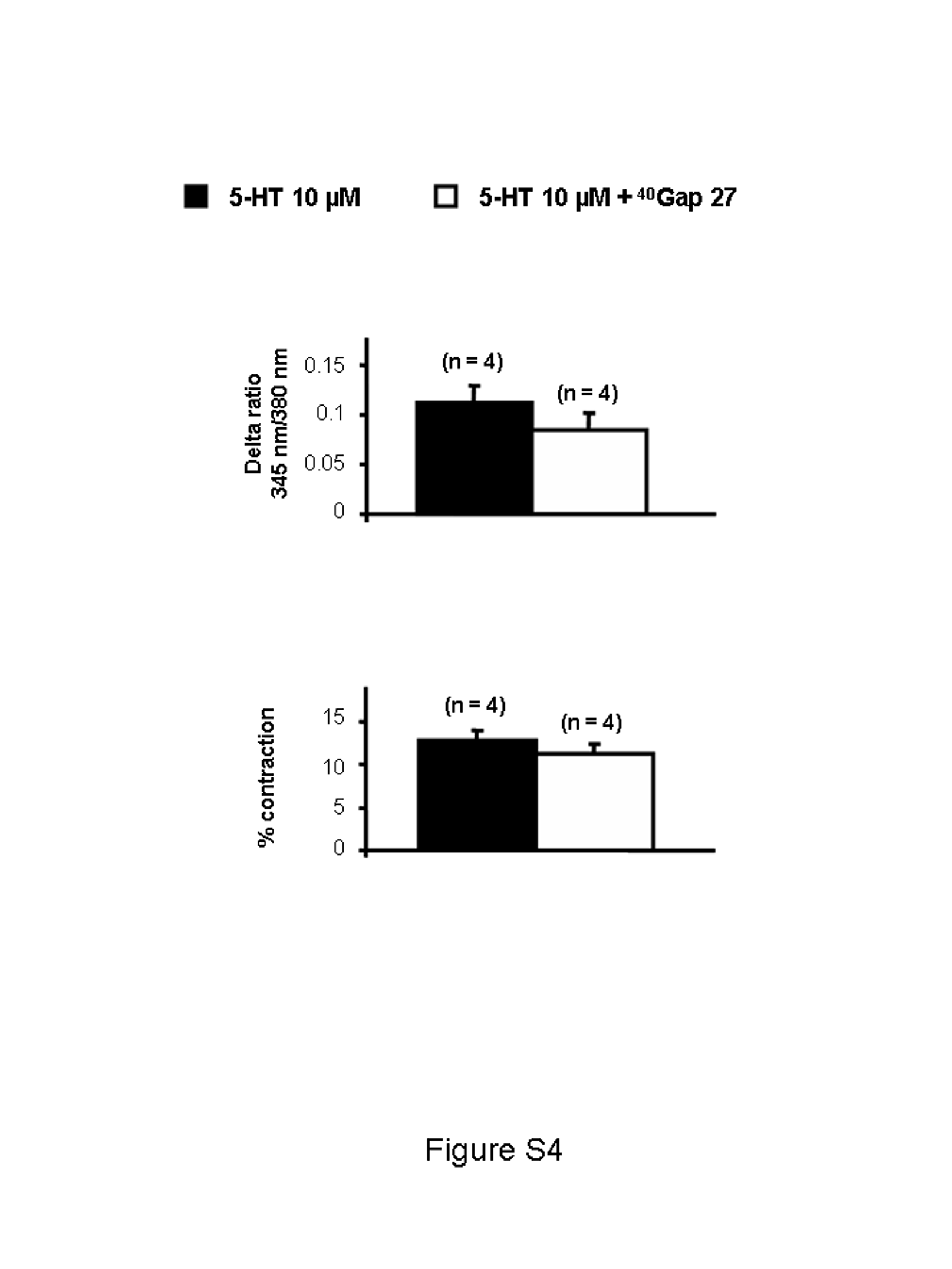

Supplement: Figure S4 — Effect of 40Gap 27 on the calcium and contractile signals in response to 5-HT. The calcium and contractile signals were simultaneously recorded in response to 5-HT 10 µM in the absence (black column) or in the presence of 300 µM 40Gap 27, the Cx-mimetic peptide targeted against Cx 40 (white column). Data are means±S.E.M. and are expressed as a delta ratio (345 nm/380 nm) for calcium signal and a percentage of contraction (top and bottom respectively). n indicates the number of vessels tested. (0.18 MB TIF) [file pone.0006432.s004.tif]

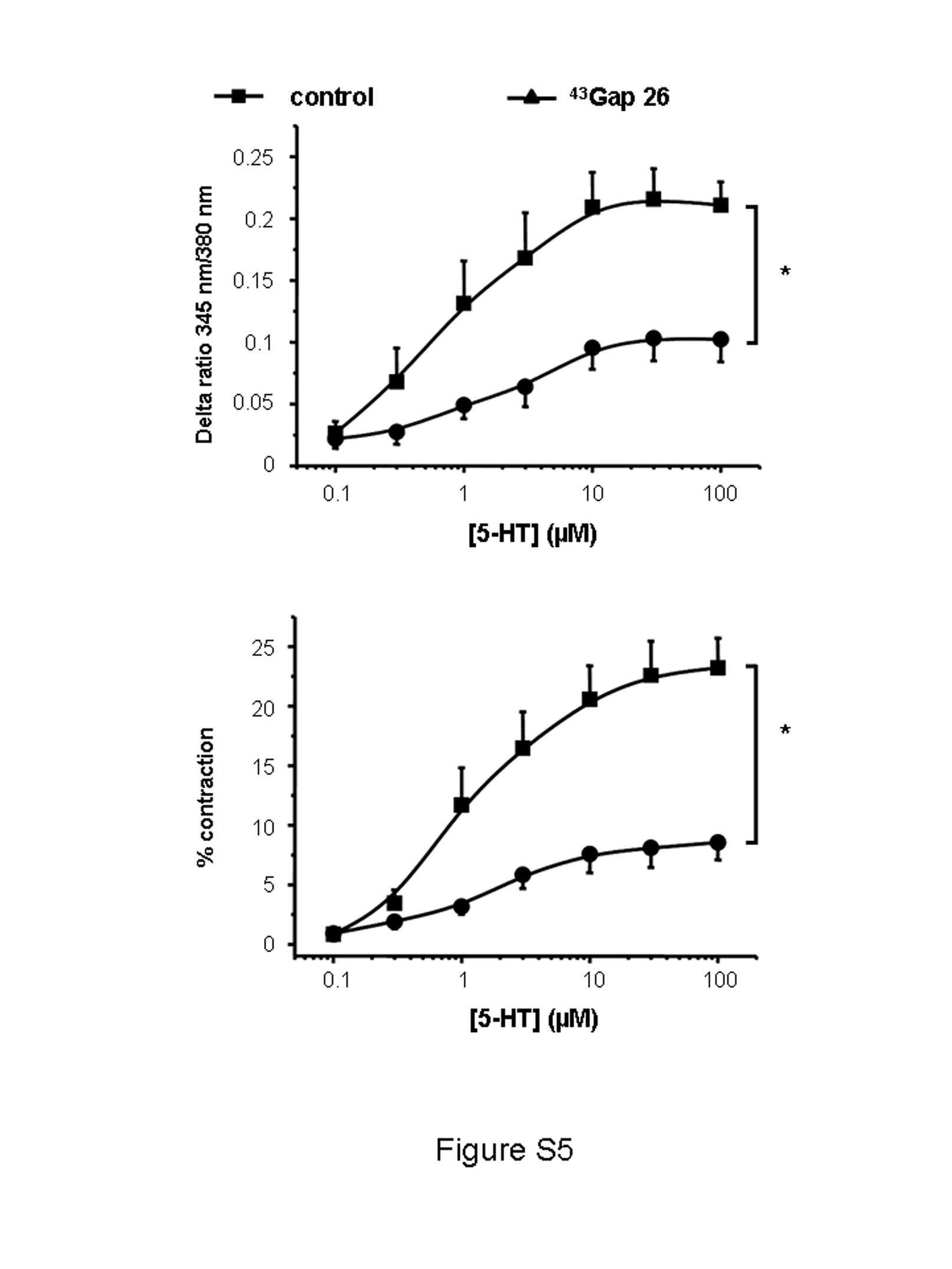

Supplement: Figure S5 — Effect of 43Gap 26 on the calcium and contractile signals in response to 5-HT. Cumulative concentration-response curves to 5-HT (0.1–100 µM) were performed in the absence or in the presence of 300 µM 43Gap 26, the Cx-mimetic peptide targeted against Cx 43 (black squares and circles respectively). Data are means±S.E.M. for 7 vessels and are expressed as a delta ratio (345 nm/380 nm) for calcium signal and a percentage of contraction (top and bottom respectively). * indicates a significant difference when P<0.05. (0.30 MB TIF) [file pone.0006432.s005.tif]

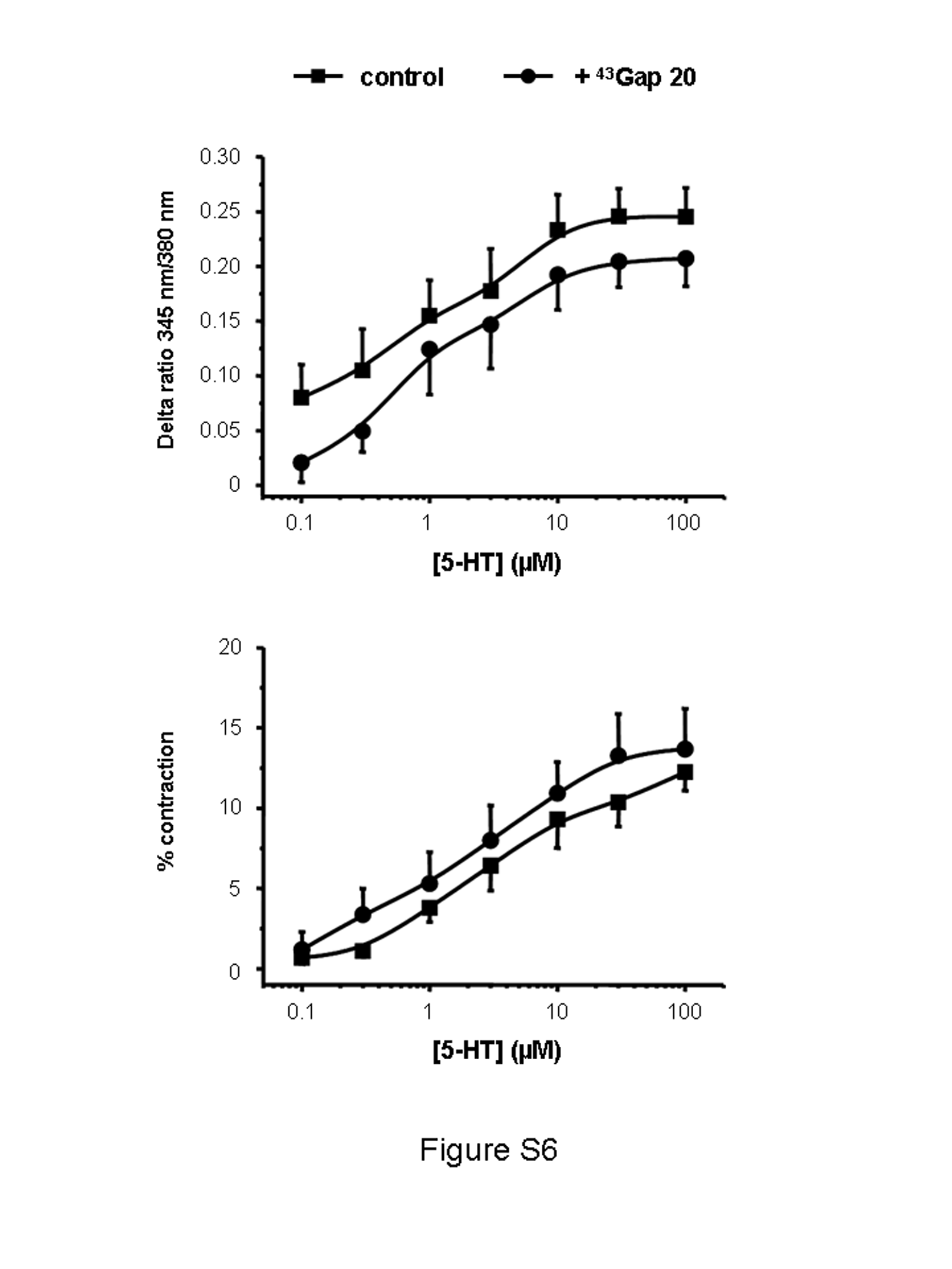

Supplement: Figure S6 — Effect of 43Gap 20 on the calcium and contractile signals in response to 5-HT. CCRC to 5-HT (0.1–100 µM) were performed in the absence or in the presence of 300 µM 43Gap 20, an inactive analog of the Cx-mimetic peptide targeted against Cx 43 (black squares and circles respectively). Data are means±S.E.M. for 7 vessels and are expressed as a delta ratio (345 nm/380 nm) for calcium signal and a percentage of contraction (top and bottom respectively). (0.30 MB TIF) [file pone.0006432.s006.tif]

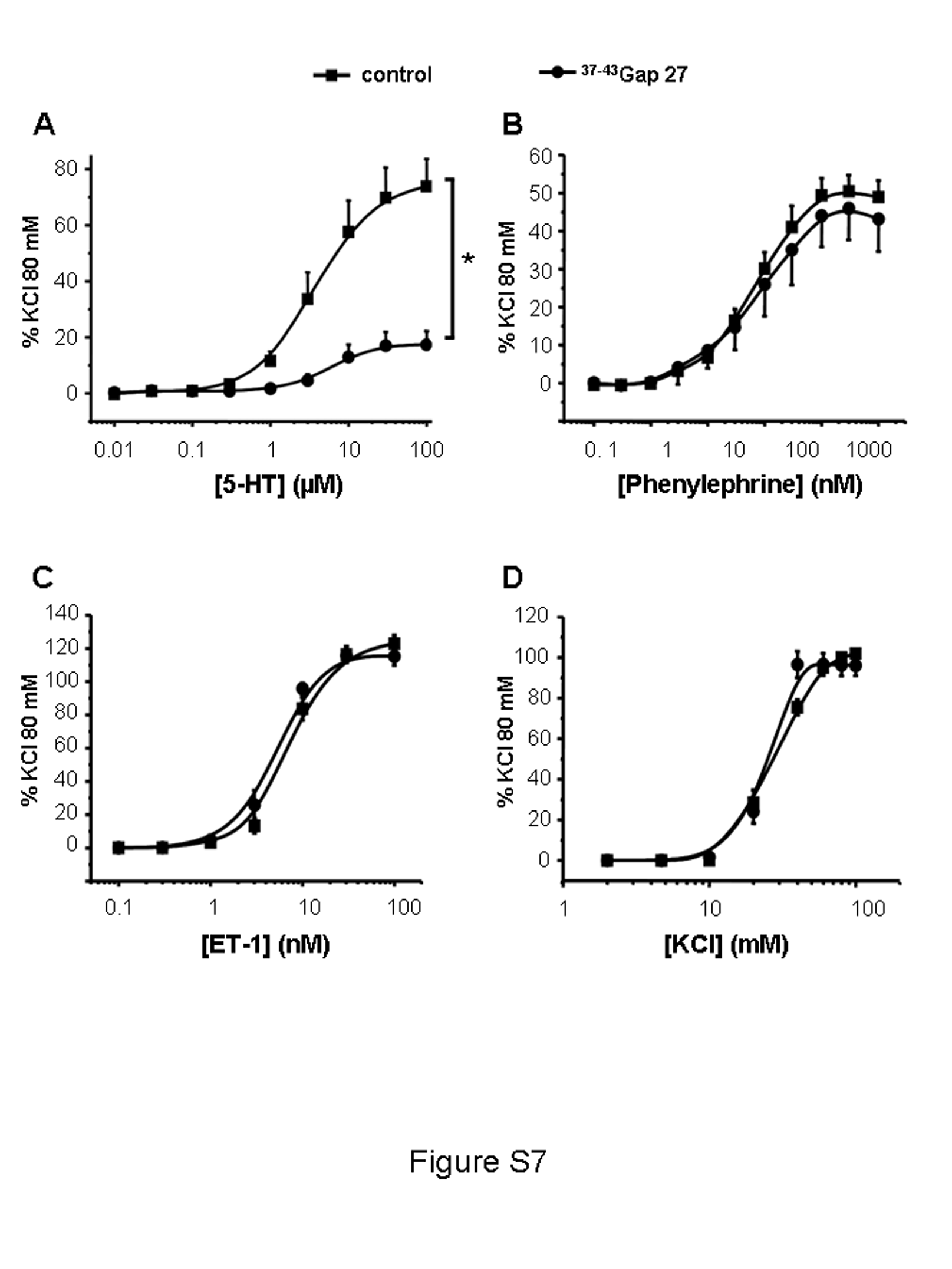

Supplement: Figure S7 — Effect of 37-43Gap 27 on the contractile responses to 5-HT, phenylephrine, endothelin-1 and high potassium solutions. Isometric tension measurements were recorded on intrapulmonary arterial rings in response to cumulative concentrations of 5-HT (A) or phenylephrine (B) or endothelin-1 (C) or in response to increasing concentrations of potassium (KCl 4.7 - 100 mM) (D). Contractions were recorded in the absence (black squares) or in the presence (black circles) of 300 µM 37-43Gap 27. Data are means±S.E.M. for 7 - 21 vessel rings and are expressed as a percentage of the contraction to high potassium solution (KCl 80 mM). * indicates a significant difference when P<0.05. (0.41 MB TIF) [file pone.0006432.s007.tif]

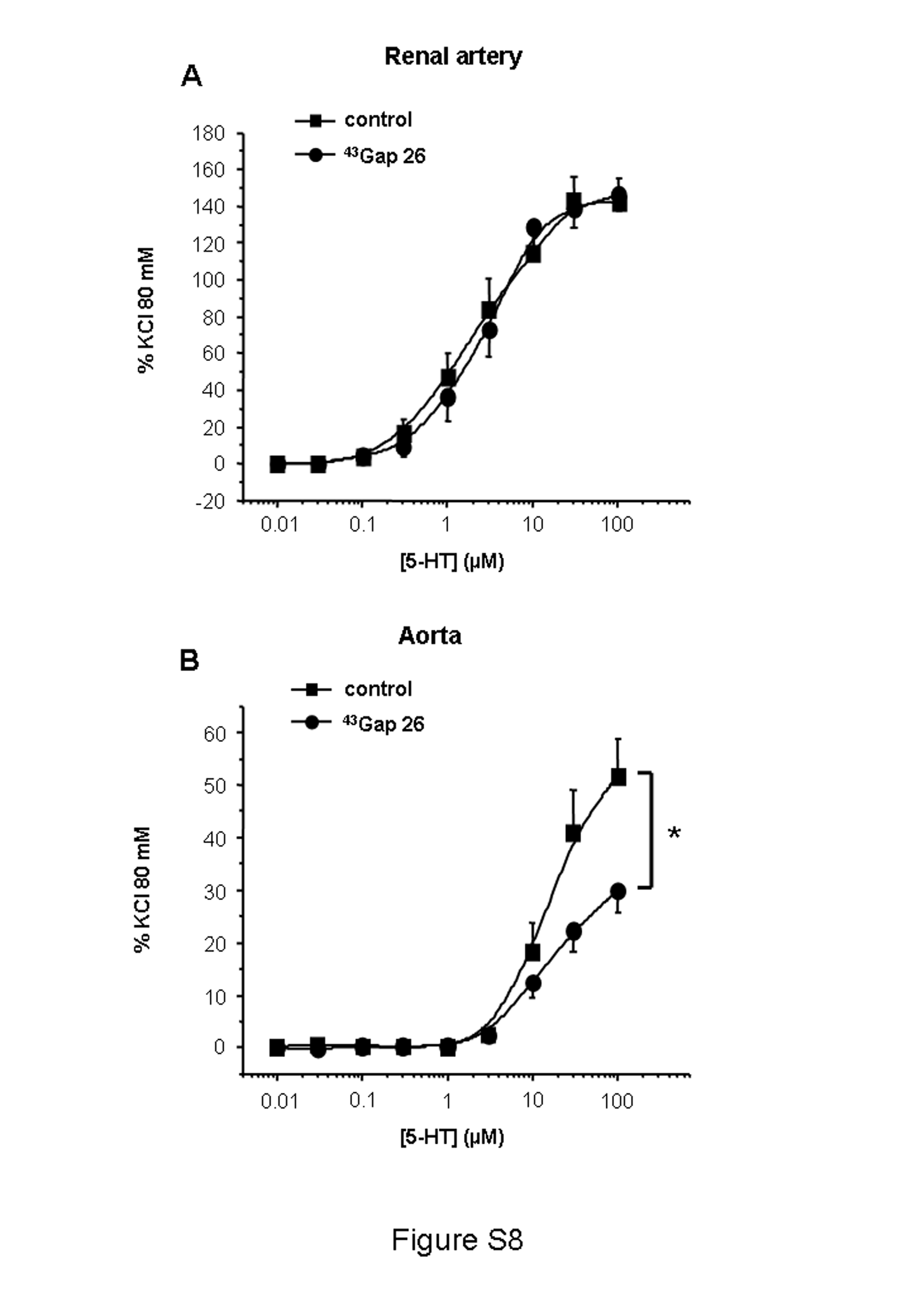

Supplement: Figure S8 — Effect of 43Gap 26 on the contractile responses to 5-HT in renal arteries and aorta. Isometric tension measurements were recorded on arterial rings in response to cumulative concentrations of 5-HT in renal arteries (A) or aorta (B). Contractions were recorded in the absence (black squares) or in the presence (black circles) of 300 µM 43Gap 26. Data are means±S.E.M. for 8 - 16 vessel rings and are expressed as a percentage of the contraction to high potassium solution (KCl 80 mM). * indicates a significant difference when P<0.05. (0.29 MB TIF) [file pone.0006432.s008.tif]

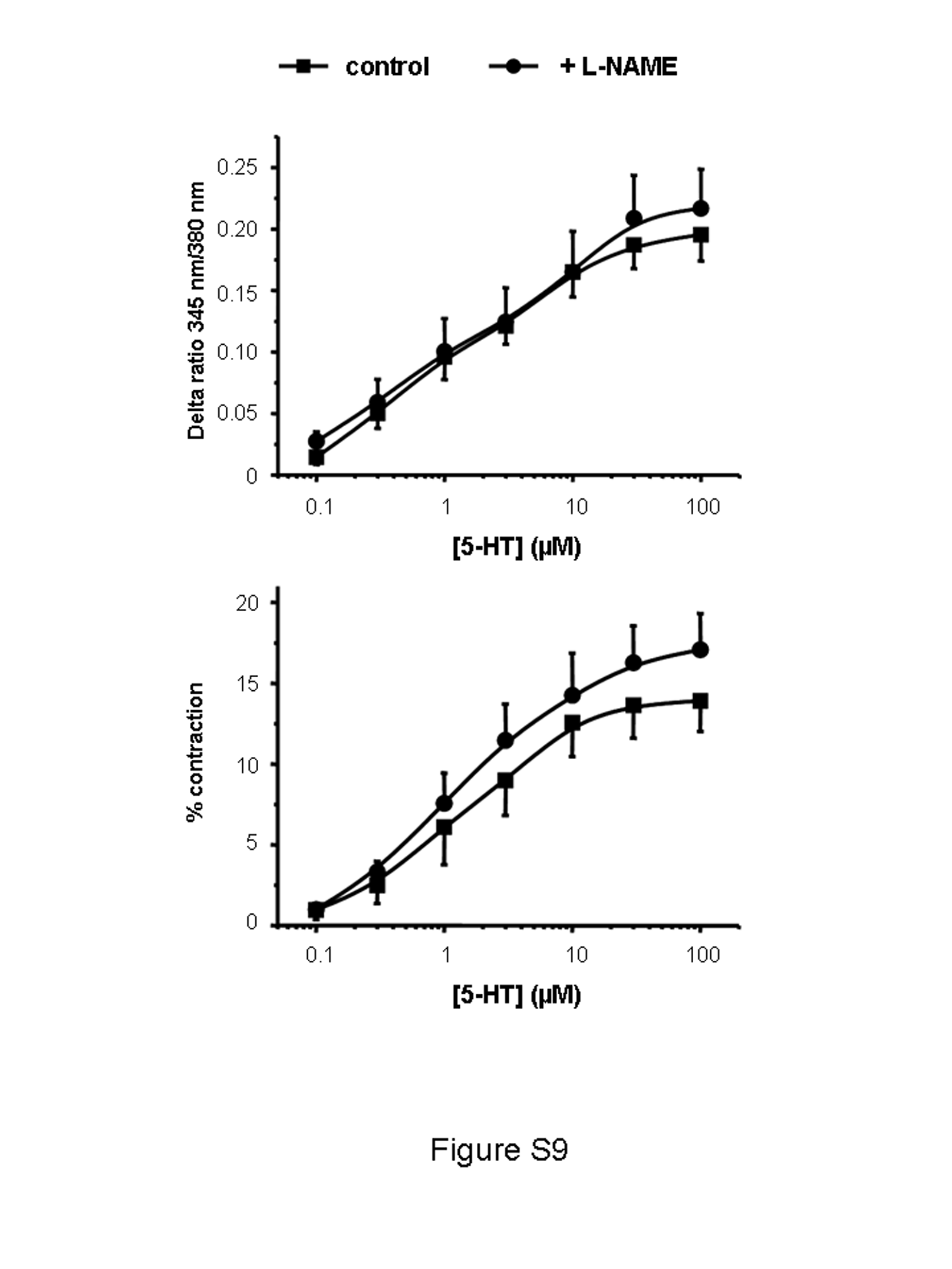

Supplement: Figure S9 — Effect of L-NAME on the simultaneously recorded calcium and contractile signals in response to 5-HT. Cumulative concentration-response curves to 5-HT (0.1–100 µM) were performed in the absence or in the presence of L-NAME 100 µM, a NO synthase inhibitor (black squares and circles respectively). Data are means±S.E.M. for 6 vessels and are expressed as a delta ratio (345 nm/380 nm) for calcium signal and a percentage of contraction (top and bottom respectively). (0.29 MB TIF) [file pone.0006432.s009.tif]

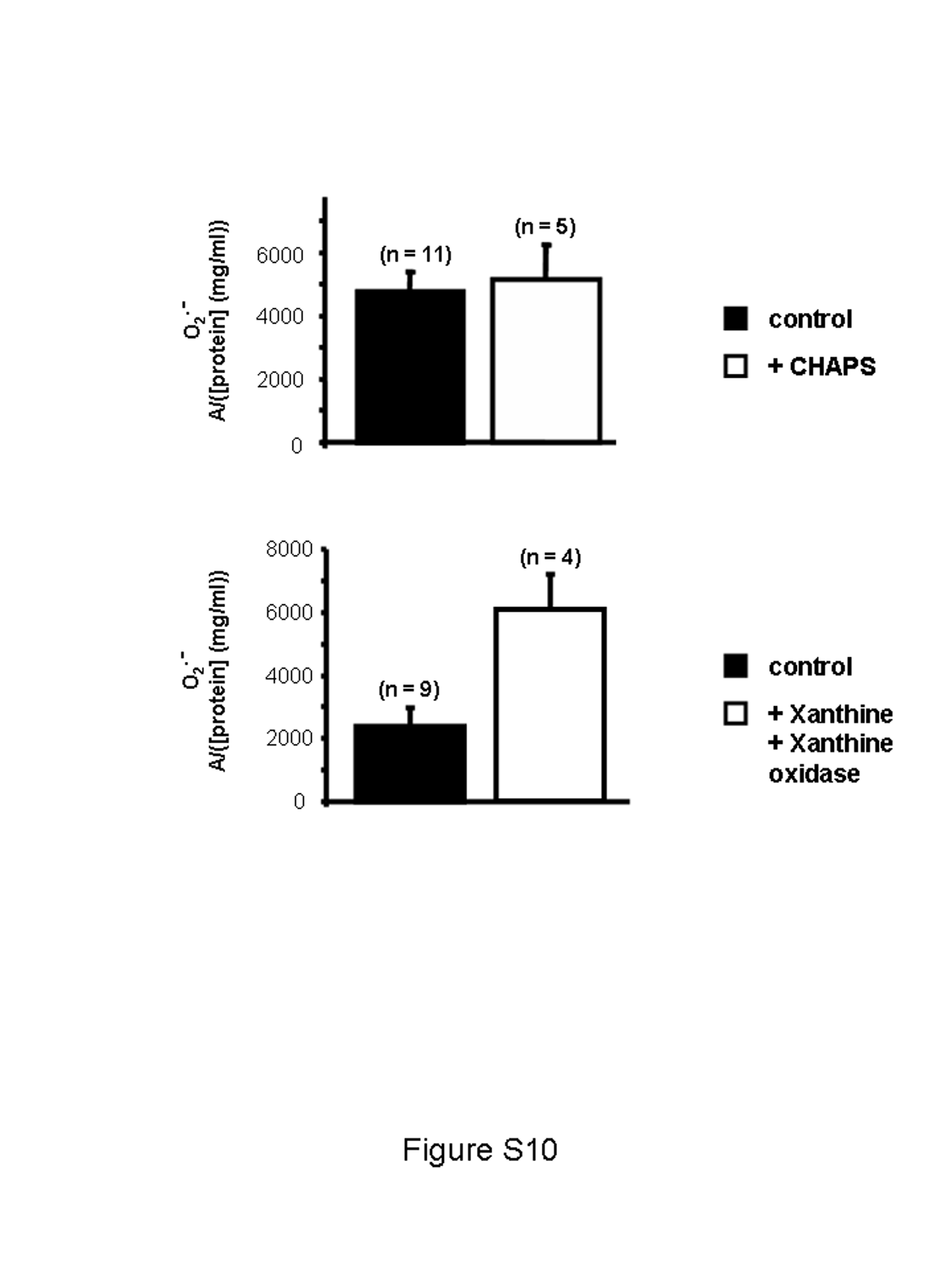

Supplement: Figure S10 — Effect of endothelium removal and xanthine plus xanthine oxidase treatment on superoxide anion production. O2• was measured by using CMH spin trapping method and EPR spectrometry on control vessels (black column), vessels whose endothelium was denuded with CHAPS 0.3% (white column, top panel) and vessels treated with xanthine 50 µM plus xanthine oxidase 0.02 U/ml (white column, bottom panel). Data are means±S.E.M. and are expressed as a ratio of the amplitude of the pic (A) out of the protein concentration of each pool of vessels in mg/ml. n indicates the number of vessels tested. (0.24 MB TIF) [file pone.0006432.s010.tif]
